# *In vitro* antimicrobial and cytotoxic evaluation of leaf, root, and stem extracts of *Solanum dasyphyllum* and root and stem extracts o*f Dovyalis abyssinica*

**DOI:** 10.3389/fphar.2025.1529854

**Published:** 2025-06-26

**Authors:** Dereilo Bekere Belitibo, Asfaw Meressa, Temesgen Negassa, Abiy Abebe, Sileshi Degu, Milkyas Endale, Frehiwot Teka Assamo, Temesgen Abdisa Ayana, Getahun Tadesse Gurmessa, Negera Abdissa

**Affiliations:** ^1^ Traditional and Modern Medicine Research and Development Directorate, Armauer Hansen Research Institute, Addis Ababa, Ethiopia; ^2^ Department of Chemistry, College of Natural and Computational Sciences, Wollega University, Nekemte, Ethiopia; ^3^ Department of Chemistry, College of Natural Sciences, Jimma University, Jimma, Ethiopia

**Keywords:** traditional medicine, antibacterial, antifungal, cytotoxic, *Solanum dasyphyllum*, *Dovyalis abyssinica*

## Abstract

**Background:**

Plants like *Solanum dasyphyllum* and *Dovyalis abyssinica* have been traditionally used to treat ailments such as fevers, asthma, high blood pressure, infections, stomachaches, skin rashes, for their blood-cleansing and anti-venom properties. Despite their traditional use, limited scientific studies have explored their pharmacological potential, particularly their antimicrobial and anticancer properties.

**Purpose:**

This study evaluated *in vitro* antimicrobial and cytotoxic activities of methanolic extracts from *Solanum dasyphyllum* (root, stem, and leaf) and *Dovyalis abyssinica* (root, stem).

**Methods:**

*Solanum dasyphyllum* and *Dovyalis abyssinica* samples were defatted using *n*-hexane and subsequently macerated in methanol to obtain crude extracts. Antimicrobial activity was assessed using agar well diffusion and micro-broth dilution methods, while cytotoxicity against the MCF-7 breast cancer cell line was evaluated using the MTT assay. Quantitative analyses of total phenolic and flavonoid contents were carried out using standard spectrophotometric assays.

**Results:**

Both plant extracts showed strong antibacterial activity, with MIC values ranging from 0.195 to 3.125 mg/mL against all tested strains. *S. epidermidis, E. coli*, and *P. aeruginosa* were especially susceptible, with MICs as low as 0.195 mg/mL for *Solanum dasyphyllum* root and stem and *Dovyalis abyssinica* root extracts. The *Solanum dasyphyllum* root extract also exhibited antifungal activity against *T. rubrum* (inhibition zone 20.67 mm). The root extract of *Dovyalis abyssinica* showed the highest cytotoxicity against MCF-7 breast cancer cells (IC_50_ = 0.67 μg/mL), outperforming doxorubicin (IC_50_ = 3.43 μg/mL).

**Conclusion:**

The methanolic extracts of both plants exhibited significant *in vitro* antibacterial, antifungal, and cytotoxic activities. Notably, the root extracts of both *Solanum dasyphyllum* and *Dovyalis abyssinica*, along with the stem extract of *S. dasyphyllum*, demonstrated strong antibacterial effects. The root extract of *D. abyssinica* showed remarkable cytotoxicity against MCF-7 breast cancer cells (IC_50_ = 0.67 μg/mL), outperforming the standard chemotherapeutic agent doxorubicin. These findings highlight its potential as a promising lead for anticancer drug development. Further studies are warranted to isolate the active constituents and confirm their therapeutic efficacy through *in vivo* investigations and clinical trials.

## 1 Introduction

Traditional medicine remains a vital component of global healthcare, particularly in developing regions where it offers a cost-effective and culturally familiar approach to managing health (Hoenders et al., 2024). Rooted in centuries of accumulated wisdom, it relies heavily on medicinal plants, which have long been used for their therapeutic properties and form the foundation of herbal medicine. This system encompasses a broad spectrum of practices, from traditional remedies to scientifically formulated herbal treatments, all of which are evaluated for safety, effectiveness, and clinical value. While deeply embedded in ancient healing traditions, herbal medicine continues to gain relevance as a complementary or alternative approach to conventional healthcare. Ongoing scientific research not only validates many of its traditional uses but also uncovers new applications, reinforcing its role within modern, integrative health systems (Balkrishna et al., 2024; Yuan et al., 2016).

In recent years, there has been a growing focus on natural products, especially from medicinal plants, as potential treatments for complex diseases like cancer. Cancer is a severe disease characterized by the uncontrolled growth of abnormal cells that invade and destroy healthy tissue which remains one of the leading causes of death worldwide, responsible for nearly 10 million deaths annually (Bray et al., 2024; Chandraprasad et al., 2021). Despite the effectiveness of conventional cancer therapies, challenges such as high costs, non-specific cytotoxicity, and the emergence of drug resistance have driven the search for alternative, more targeted treatments. Natural products, particularly plant-derived compounds, offer promising anticancer potential by targeting various molecular pathways involved in cancer progression, such as apoptosis, oxidative stress, and angiogenesis (Lo Iacono et al., 2021; Kudamba et al., 2023). In fact, over 60% of current anticancer drugs are derived from natural sources, with plant-based compounds like paclitaxel, vincristine, and camptothecin serving as prime examples (Pezzuto, 1997; Newman and Cragg, 2020). As research advances, increasing attention is being paid to the isolation of bioactive compounds and the understanding of their molecular mechanisms, which has further highlighted the therapeutic potential of medicinal plants, not only for cancer but also for their anti-inflammatory, and antimicrobial effects (Sharifi-Rad et al., 2022; Zhao et al., 2022).

In parallel, infectious diseases caused by the global rise of multidrug-resistant bacteria (MDRB) pose a serious public health threat, contributing significantly to morbidity and mortality and posing challenges to health security, particularly in low- and middle-income countries. The overuse and misuse of antibiotics have accelerated the evolution of resistant strains, reducing treatment efficacy and increasing the burden of infectious diseases (Ventola, 2015; Nii-Trebi, 2017). Methicillin-resistant *Staphylococcus aureus* (MRSA), for instance, is a leading cause of hospital- and community-acquired infections worldwide. In response, the scientific community is turning to plant-based antimicrobials as potential alternatives (Abebe and Birhanu, 2023). Phytochemicals such as phenolic acids, tannins, and flavonoids have shown efficacy against resistant pathogens by disrupting microbial membranes, inhibiting enzyme activity, and modulating quorum sensing (Zorzan et al., 2020; Shahrajabian and Sun, 2023; de Kraker et al., 2016). In addition to their antimicrobial effects, compounds present in these plants also exhibit immunomodulatory and protective properties, contributing to enhanced immune responses and reduced risk of infections and chronic disease progression (Alfuraydi et al., 2024; Pizzino et al., 2016).

**FIGURE 1:**
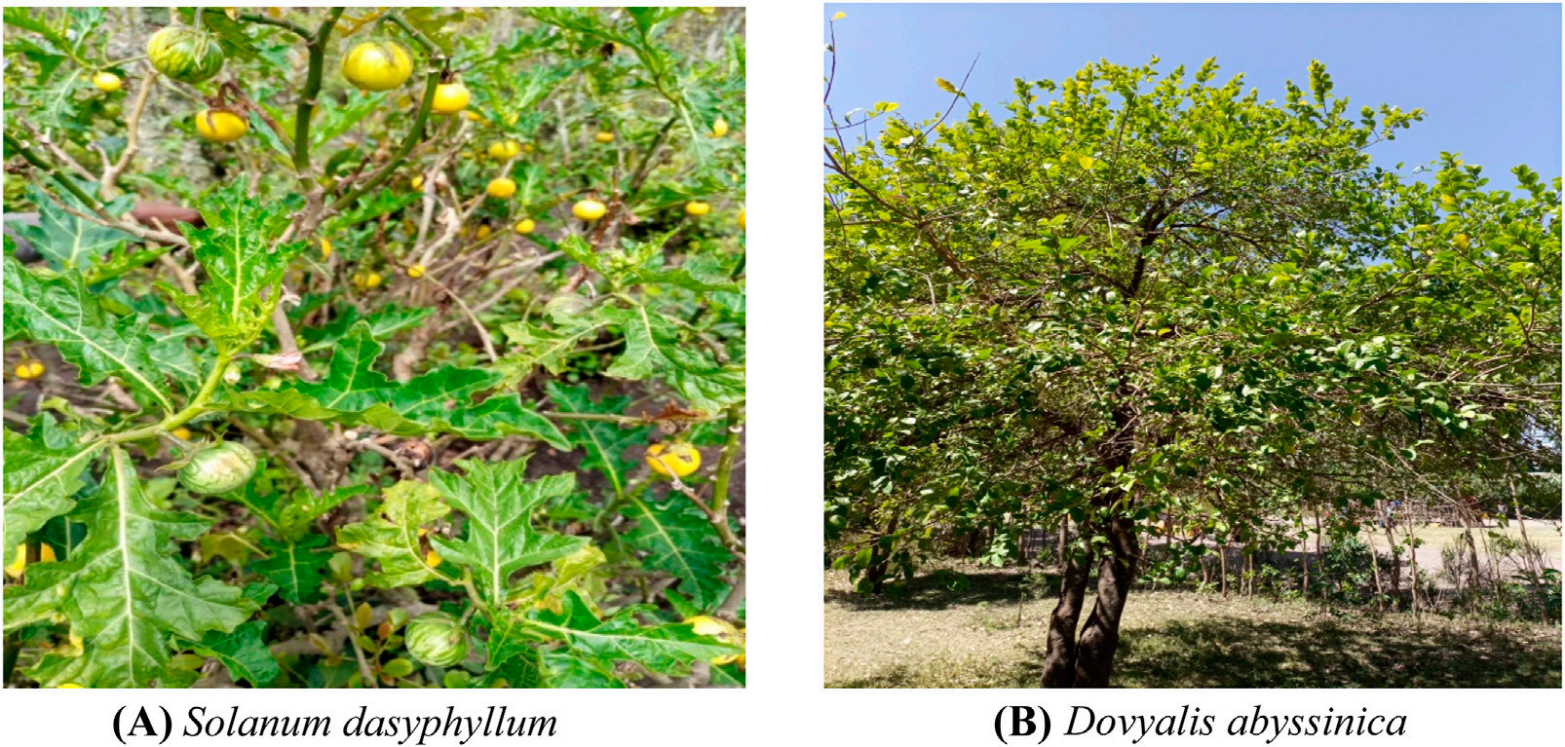
Whole Plant picture of **(A)** *Solanum. dasyphyllum* and **(B)** *Dovyalis abyssinica* (Photograph by Dereilo Bekere, October 2024).

Among the diverse array of medicinal plants, *Dovyalis abyssinica* A. Rich Warb (Salicaceae family) (Figure 1B) and *Solanum dasyphyllum* Schumach. & Thonn. (Solanaceae family) (Figure 1A) are ethnomedicines, representing traditional alternative treatments with a range of benefits. These remedies, native to Ethiopia, have long been employed by traditional healers for various ailments, offering cost-effective solutions with minimal cellular side effects and easy management (Beyi, 2018; Enyew et al., 2014; Funmilola et al., 2020; Kigen et al., 2017; Njoroge and Bussmann, 2009; Tuasha et al., 2018). *Solanum dasyphyllum’s* root is utilized for countering poisons from venomous stings and bites (Funmilola et al., 2020), as well as relieving abdominal colic (Paulos et al., 2016). Its leaves find application in treating gastrointestinal issues, gout, and inflammation (Oyinloye et al., 2022), while the fruits are employed to combat external parasites (Kitata et al., 2017). Additionally, the leaf sap and flowers serve as remedies for subcutaneous parasitic infections and as antidotes in cases of *Strophanthus* poisoning (Sodeinde et al., 2019). Likewise, *D. abyssinica*’s roots are employed for alleviating stomachaches, fevers (Amuka et al., 2014), reproductive health issues (Njoroge and Bussmann, 2009), epilepsy (Kigen et al., 2017), and rheumatic pain (Beyi, 2018). The stem bark, on the other hand, is utilized in treating cancerous tumors (Abate et al., 2022; Tuasha et al., 2018) and ascaris infections (Beyi, 2018). Its leaves are used to address body swelling (Bogale et al., 2023), regulate blood pressure, alleviate asthma (Alemneh, 2020), and manage cold-related symptoms (Eshete et al., 2016). In addition, the fruits of *D. abyssinica* are administered for relieving abdominal pain (Mazengia et al., 2019), Abdominal Helminthes, Parasites and combating cancer (Enyew et al., 2014), while the seeds are utilized in treating hemorrhoids (Bogale et al., 2023). Furthermore, the Whole parts of *D. abyssinica* are administered for Swelling (Ayele, et al., 2024).

Phytochemical investigations of S. *dasyphyllum* and *D. abyssinica* have demonstrated that both plants are rich in bioactive constituents, including phenolics, flavonoids, and alkaloids (Oyinloye et al., 2023; Chirchir et al., 2014). Although research is still limited, existing pharmacological studies have reported several promising biological activities. These include antimicrobial effects (Oyinloye et al., 2023; Chirchir et al., 2014; Legesse et al., 2019), antihyperglycemic properties and wound healing potential (Baylie et al., 2024; Abate et al., 2022), as well as anti-inflammatory (Oyinloye et al., 2021; Abdissa, 2011), anti-trypanosomal, neuroprotective, and anti-venom activities (Ilesanmi et al., 2020; Funmilola et al., 2020). Despite their extensive use in traditional medicine and the presence of promising phytochemical profiles, both species remain underexplored in terms of rigorous scientific validation. The existing evidence is fragmented and insufficient to fully understand their therapeutic potential, underscoring the need for more comprehensive pharmacological studies.

In this context, the present study aims to evaluate the antimicrobial and cytotoxic properties of methanolic extracts from the root, stem, and leaf of *S. dasyphyllum* and the root and stem of *D. abyssinica*. This study seeks to bridge ethnomedicinal knowledge with experimental evidence and highlight the therapeutic promise of these underexplored species.

## 2 Materials and methods

### 2.1 General experimental procedures

Analytical and HPLC grade solvents and reagents were used for extraction. For analysis, Whatman No.1 filter paper (Whatman Ltd. International, Maidstone, UK), Tween, Petri dishes, cork borer with diameter of 8 mm, pre-labelled 96- well micro titter-plate, Biosafety Cabinet (Telstar), UV-Visible Spectrophotometer (Thermo Scientific Evolution 60S), Incubator (Memmert Germany), Whirl mixer (borfax), Mueller Hinton Broth (MHB) (Sigma Aldrich), Sabouraud Dextrose Broth (SDB) (Sigma-Aldrich), Ciprofloxacin Refrance standard (Germany-Twinbrook pkwy), Amphotericin-B reference standard (Italy, Twinbrook pkwy) and 2-3,5-Triphenyltetrazolium chloride (TTC) (Sigma Aldrich) were used. Dulbecco’s Modified Eagle Medium Nutrient Mixture F-12 (DMEM F12) from Gibco (United States), Fetal Bovine Serum Heat Inactivated (FBS) from HiMedia (India), Antibiotic Slution 100X (10,000U Penicillin and 10 mg Streptomycin per ml) from HiMedia (India), Thiazolyl Blue Tetrazolium Bromide (MTT) from SRL (India).

### 2.2 Plant materials collection

The roots, stems, and leaves of *Solanum dasyphyllum* and the roots and stems of *Dovyalis abyssinica* were collected in December 2021 from East Badewacho Woreda in the Hadiya Zone of central Ethiopia, near Shone town (7.14421°N, 37.95558°E, at an elevation of 1,958 m). The site is approximately 338 km from Addis Ababa via Shashimane and 98 km from Hosanna Town. The plant species were identified by Prof. Sileshi Nemomisa, a botanist, through herbarium comparison. The botanical specimens were then deposited at the National Herbarium of Addis Ababa University, Ethiopia, with voucher specimen numbers DB-01 for *Solanum dasyphyllum* and DB-05 for *Dovyalis abyssinica*. The plants (Figure 1) were selected for their ethnopharmacological uses, specifically for their antimicrobial and anticancer properties, based on interviews with local communities and traditional healers.

### 2.3 Plant materials preparation and extraction

The collected plant materials were air-dried in a shaded area to avoid direct sunlight and then ground into a fine powder using a mortar and pestle. A total of 0.5 kg of the coarse plant powder was first defatted with n-hexane at a ratio of 1:5 (v/w) in Erlenmeyer flasks. These flasks were placed on an orbital shaker and left to shake for 24 h at a speed of 130 revolutions per minute. This process was repeated three times to remove non-polar compounds such as lipids, oils, and waxes before the next stage of extraction. After each 24-h period, the solution was filtered through 15-cm Whatman No. 1 filter paper. The residue was then dried and soaked in methanol at a ratio of 1:5 (v/w) using the same procedure. The methanol extraction was repeated three times to maximize the yield. The filtered solution was concentrated using a rotary evaporator (Buchi, R-300, Switzerland) at 40°C to obtain the crude extract. The resulting crude sample was transferred to various petri dishes, labeled with sample identifiers, and left in a fume hood for several days until fully dried. Once dried, the samples were stored in a refrigerator for further analysis. This procedure was applied to both plant parts, yielding 22 g (4.4%) from the root, 19 g (3.8%) from the stem, and 24 g (4.8%) from the leaf of *S. dasyphyllum*, as well as 26 g (5.2%) from the root and 23 g (4.6%) from the stem of *D. abyssinica*.

### 2.4 Determination of total phenolic and flavonoid content of the extracts

#### 2.4.1 Determination of total phenolic content (TPC)

The total phenolic content (TPC) in the plant extracts was determined using a modified Folin–Ciocalteu colorimetric method (Singleton et al., 1999; Ramli A. N. M. et al., 2020). A standard gallic acid solution was prepared by dissolving 10 mg of gallic acid in 10 mL of methanol (1 mg/mL). Gallic acid solutions at concentrations of 25, 50, 75, and 100 μg/mL were then prepared from this stock solution. To each concentration, 5 mL of 10% Folin–Ciocalteu reagent and 4 mL of 7% Na_2_CO_3_ were added, bringing the final volume to 10 mL. The resulting blue-colored mixture was incubated at room temperature for 120 min, after which the absorbance was measured at 760 nm against a blank. The FCR reagent oxidizes phenols in the plant extracts, producing a dark blue color, which was then quantified using a UV-visible spectrophotometer. The total phenolic content was expressed as milligrams of gallic acid equivalents (GAE) per gram of dried extract. All experiments were conducted in triplicate, and the average absorbance values at various gallic acid concentrations were used to construct the calibration curve.

#### 2.4.2 Determination of total flavonoid content (TFC)

The total flavonoid content was determined using the aluminum chloride colorimetric assay (Bunea, et al., 2011; Ramli A. N. M. et al., 2020; Bhuyar et al., 2020). A quercetin standard solution was prepared with concentrations ranging from 0 to 80 μg/mL, and a calibration curve was established. The samples were diluted with DMSO and made up to 50 mL with methanol. To each sample, 4 mL of distilled water, 1 mL of the sample or standard solution, and 300 µL of 5% NaNO_2_ were added to a glass cuvette. After 5 min, 300 µL of 10% AlCl_3_ was added, followed by 2 mL of 1M NaOH and 2.4 mL of distilled water after 6 min. The mixture was incubated for 10 min at room temperature. Each experiment was performed in triplicate, and the absorbance was measured at 510 nm using a UV spectrophotometer. The total flavonoid content was expressed as milligrams of quercetin equivalents (QE) per gram of dry extract. The results are presented as the mean ± SD of three replicates.

### 2.5 Pathogenic bacterial and fungal strains

The antibacterial activity of methanol extracts of *S. dasyphyllum* root, stem, and leaf and *D. abyssinica* root and stem were evaluated against eight pathogenic standard bacteria and fungi American Type Culture Collection (ATCC) and one clinical isolate Yeast *Candida albican*. The tested pathogenic bacteria were three Gram-positive (*Staphylococcus aureus* (ATCC 25923), *Staphylococcus epidermidis* (ATCC 12228), and *Enterococcus faecalis* (ATCC 29212) and three Gram-negative *Escherichia coli* (ATCC 25922), *Klebsiella pneumoniae* (ATCC 700603) and *Pseudomonas aeruginosa* (ATCC 27853), and three pathogenic fungi (*Trichophyton mentagrophytes* (ATCC 18748), *Trichophyton rubrum (ATCC 28188)*, and one clinical isolate *Candida albican* were obtained from Armauer Hansen Research Institute (AHRI) for antimicrobial activities. These microorganisms were maintained in the Traditional and Modern Medicine Research and Development Directorate (TMMRDD) Microbiology Laboratory of AHRI in Triptic soya broth +20% glycerol (bacteria) or Sabouraud Dextrose Broth (SDB) + 20% glycerol (fungi) at −78°C.

### 2.6 Inoculum preparation

The inoculum size of test microbe bacteria and fungi were standardized according to Clinical and Laboratory Standards Institute Guidelines (Wayne, 2011). From stored stock cultures, the bacteria were refreshed in Petri dishes containing nutrient agar by incubating for 18–24 h at 37°C. Similarly, fungi were refreshed by sub-culturing in Petridishes containing Sabouraud dextrose agar medium by incubating at 25°C for up to 7 days. For the actual test active cultures were prepared from stored and refreshed cultures at 4°C, subcultured at 37°C overnight on Mueller Hinton Agar (Sigma-Aldrich), and up to 7 days at 25°C on Sabouraud Dextrose Agar (SDA) (Sigma-Aldrich) for both bacterial and fungal test organisms, respectively. Test organisms were standardized by diluting with broth medium and measuring their absorbance using a spectrophotometer adjusted at 625 nm with an absorbance reading of OD value range from 0.08 to 0.1, which is equivalent to 1.0 × 10^8^ CFU/mL for bacteria and 1.0 × 10^7^ CFU/mL for fungal species, respectively. The suspensions were further 1:10 diluted with respective broth to 1.0 × 10^7^ CFU/ml and 1 × 10^6^ CFU/mL for final inoculum size of bacteria and fungi, respectively (Al-Salt, 2012; Huse et al., 2018).

### 2.7 Evaluation of the antimicrobial activity

The antibacterial and antifungal activities of the methanol extracts of *S. dasyphyllum* (roots, stems, and leaves) and *D. abyssinica* (roots and stems) were tested against six pathogenic bacteria (three Gram-positive and three Gram-negative) and three fungal strains using agar well diffusion and Broth micro-dilution (minimum inhibitory concentration) for MIC assays as described in the literature (Eloff, 1998; Humphries et al., 2021; Weinstein and Lewis, 2020).

#### 2.7.1 Antimicrobial activity screening

The agar well diffusion method was used to evaluate the antimicrobial activity of the crude extracts. Solidified agar plate surface was inoculated uniformly by spreading with sterile cotton swab by briefly dipping into the standardized and adjusted microbial inoculum suspensions, over the entire agar surface. Then, hole with a diameter of 8 mm has punched aseptically with a sterile cork borer, and a volume 100 μL of the antimicrobial agent or extract solution at the desired concentration (100 mg/mL and 200 mg/mL) was introduced into the well. Then, the plates were incubated under suitable conditions at 37°C for 18–24 h for antibacterial activity and at 25°C up to 7 days for the antifungal activity. The antimicrobial agent diffuses in the agar medium and inhibits the growth of the tested microbe. 5% tween 80 in sterile distilled water used as negative control and ciprofloxacin (5 μg/mL) (Germany, Twinbrook pkwy), Amphotericin B (32 μg/mL) (Italy, Twinbrook pkwy) were used as positive controls for both bacteria and fungi, respectively. All tests were performed in triplicate, pararally with growth and sterility controls and the zone of inhibition was measured using millimeter of ruler (Al-Salt, 2012; Huse et al., 2018).

#### 2.7.2 Minimum inhibitory concentration

All tested extracts were analyzed to determine their minimum inhibitory concentration (MIC) using the micro-broth dilution method. Various concentrations of the extracts were prepared, and 100 µL of broth medium was added to the first row of a 96-well microplate. Using a multichannel micropipette, two-fold serial dilutions were carried out from row A to row H, resulting in extract concentrations ranging from 25 mg/mL to 0.195 mg/mL. Subsequently, 100 µL of bacterial inoculum (5 × 10^5^ CFU/mL) was added to each well within 10–15 min after standardizing the bacterial suspension. The plates were then incubated at 37°C for 18–24 h. After the incubation period, bacterial growth at each extract concentration was assessed. To confirm the presence of microbial growth, 40 µL of a 0.4 mg/mL solution of 2, 3, 5-triphenyltetrazolium chloride (TTC) was added to each well, and the plates were re-incubated for 30 min at 37°C. The formation of a purple color, indicative of bacterial growth, was visually assessed using magnifying instruments to determine the MIC values. All assays were performed in triplicate, as described in the literature (Mostafa et al., 2018). Several quality control measures were performed in parallel to ensure the accuracy of the experiments. These included negative controls with 5% Tween 80, positive controls with ciprofloxacin, sterility controls, and growth controls. The MIC value was defined as the lowest concentration of the extract that inhibited bacterial growth under the specified incubation conditions, and the results were expressed in mg/mL (Eloff, 1998; Weinstein and Lewis, 2020).

### 2.8 *In vitro* cytotoxic evaluation against cancer cell lines

The *in vitro* cytotoxic effect of the test plant extracts was evaluated using the MTT assay (Mosmann, 1983; Stockert et al., 2012). Breast cancer cells (MCF7), obtained from the National Center for Cell Science (NCCS), Pune, India, were cultured in DMEM F12 (Dulbecco’s Modified Eagle Medium/Nutrient Mixture F-12) medium supplemented with 10% FBS and 1X Penicillin/Streptomycin solution at 37°C in a humidified atmosphere with 5% CO_2_. Once the cells reached confluency, they were trypsinized, passaged, and prepared for further assays. A stock solution of 1 mg/mL of each plant extract was prepared in methanol. Treatment media were prepared with concentrations of 50 μg/mL, 100 μg/mL, and 200 μg/mL of *S. dasyphyllum* and *D. abyssinica* extracts, with Doxorubicin used as a positive control. The MCF7 cells were seeded in triplicate at a density of 10,000 cells/well in 96-well plates with DMEM F12 medium supplemented with 10% FBS and 1X Penicillin/Streptomycin solution. When the cells reached approximately 70% confluence, they were treated with the prepared treatment media. After 24 h of treatment, the MTT assay was performed. The spent media was removed, and 10 µL/100 mL of MTT reagent (5 mg/mL stock) was added. The plates were incubated for 4 h at 37°C, after which the MTT reagent was replaced with DMSO to dissolve the formazan crystals. The absorbance was measured at 570 nm using an ELISA plate reader. All experiments were conducted in triplicate.

$$\%\text{ Cell Viability}=\left(S/C\right)\times 100$$

% Inhibition=100‐% Cell viability

Where, S = Optical density (OD) of the sample, C = Optical density (OD) of the control.

### 2.9 Spectral and statistical data analyses

All experimental data were statistically analyzed using SPSS version 16 and Microsoft Excel. One-way ANOVA was applied to assess differences in antibacterial, antifungal, and *in vitro* cytotoxic responses. Each experiment was conducted in triplicate, and results were reported as mean ± standard deviation (SD). Antimicrobial efficacy was expressed as mean MIC values and/or zones of inhibition. Cytotoxicity was reported as percentage cell viability. All statistical results were considered significant at a 95% confidence level (P < 0.05).

## 3 Results and discussion

### 3.1 Total phenolic and flavonoid content of the extracts

#### 3.1.1 Total phenolic contents (TPC)

The total phenolic content (TPC) in extracts from *S. dasyphyllum* (root, stem, and leaf) and *D. abyssinica* (root and stem) was quantified using the Folin–Ciocalteu method, with gallic acid serving as the standard reference. A calibration curve was constructed using a series of gallic acid concentrations, and the TPC values were expressed as milligrams of gallic acid per gram of crude extract (Table 1). The TPC values observed for the extracts of both plants ranged from 77.50 to 143.22 mg gallic acid per gram. Among the different extracts, the highest TPC was recorded in the methanolic extract of *D. abyssinica* root, which contained 143.22 ± 0.317 mg gallic acid/g. This was followed by the root extract of *S. dasyphyllum*, which had a TPC of 136.3 ± 0.451 mg gallic acid/g. The lowest TPC value was observed in the methanolic extract of *D. abyssinica* stem, which had a TPC of 77.50 ± 0.265 mg gallic acid/g. These findings indicate that both plant species, particularly their methanolic extracts, are rich sources of phenolic compounds.

**TABLE 1:** Total phenolic and flavonoids contents, of metanolic extracts of *S. dasyphyllum* and *D. abyssinica*.

Sample/Extract type		Total phenolic content (mg gallic acid/g)	Total flavonoids content (mg catechin/g)
*S. dasyphyllum*	root	136.3 ± 0.451^a^	90.23 ± 0.315^d^
	stem	114.29 ± 0.977^b^	59.42 ± 0.643^f^
	leaf	98.25 ± 0. 444^c^	18.33 ± 0.788^g^
*D. abyssinica*	root	143.22 ± 0.317^a^	112.36 ± 0.249^b^
	stem	77.50 ± 0.265^e^	76.67 ± 0.217^e^

Different lowercase superscript letters within each column indicate statistically significant differences among fractions (p < 0.05). Error bars represent ±SD of three replicates.

#### 3.1.2 Total flavonoid contents (TFC)

The total flavonoid content (TFC) in the plant extracts was determined using the aluminum chloride method, with catechin as the reference standard. A calibration curve was prepared using different concentrations of catechin, and the TFC values were expressed as milligrams of catechin per gram of crude extract. The TFC values followed a similar trend to those of the total phenolic content (TPC), with the values in both plant extracts ranging from 18.33 to 112.36 mg catechin per gram. The highest TFC was observed in the methanolic extract of *D. abyssinica* root, which had a value of 112.36 ± 0.249 mg catechin/g, followed by the root extract of *S. dasyphyllum* at 90.23 ± 0.315 mg catechin/g. The lowest TFC was found in the leaf extract *of S. dasyphyllum*, which had a value of 18.33 ± 0.788 mg catechin/g. These results indicate that the methanolic extracts of both plants are rich in flavonoids, as shown in Table 1.

### 3.2 Antimicrobial activities results

#### 3.2.1 Antibacterial activity

##### 3.2.1.1 Antibacterial activity screening by the agar-well diffusion method

**FIGURE 2:**
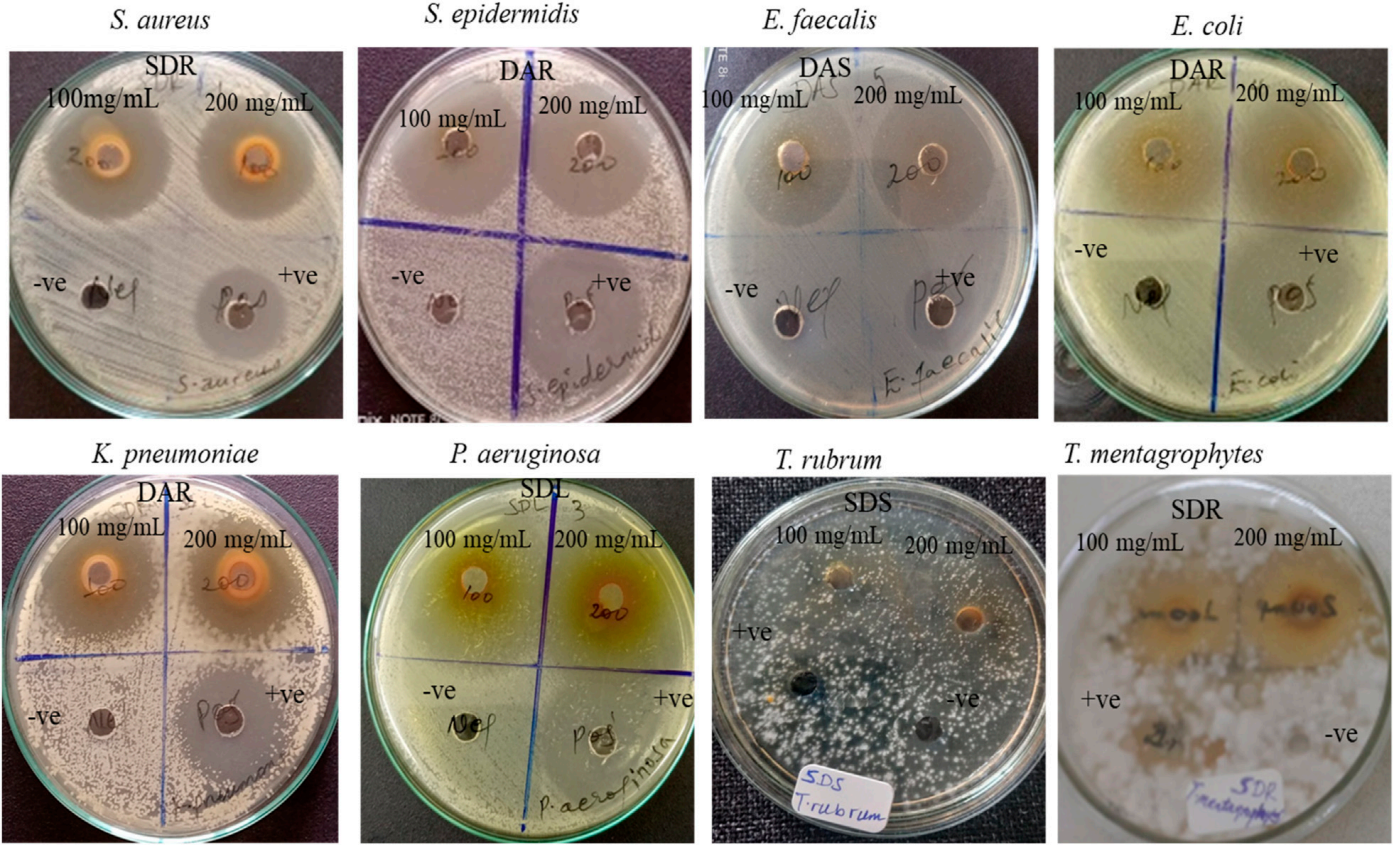
Examples of Antimicrobial activity of *S. dasyphyllum* and *D. abyssinica* extracts from soot, stem, and leaf were tested using agar diffusion against *S. aureus, S. epidermidis, E. faecalis, E. coli, K. pneumoniae, P. aeruginosa, C. albicans, T. mentagrophytes*, and *T. rubrum* at 100 and 200 mg/mL concentrations. Key notes: Extract type SDR= *S. dasyphyllum* root, SDS= *S. dasyphyllum* stem, SDL= *S. dasyphyllum* leaf, DAR=*D. abyssinica* root and DAS=*D. abyssinica* stem.

The antibacterial activity results of *S. dasyphyllum* and *D. abyssinica* methanolic extracts reveal potent and broad-spectrum inhibitory effects against both Gram-positive and Gram-negative bacteria. This suggests that these traditionally used medicinal plants harbor significant antibacterial properties, potentially due to the presence of bioactive phytochemicals such as flavonoids, alkaloids, phenolic acids, and tannins. The extracts were tested against a panel of clinically relevant bacterial strains: *S. aureus*, *S. epidermidis*, and *E. faecalis* (Gram-positive); and *E. coli*, *K. pneumoniae*, and *P. aeruginosa* (Gram-negative), at concentrations of 100 and 200 mg/mL. The agar-well diffusion assay results indicate that all tested plant parts exhibited inhibitory activity, with variation in potency depending on plant species, specific plant part, and bacterial strain (Table 2; Figure 2).

**TABLE 2:** Bacterial growth inhibition zone diameter measurement in (mm) of *S. dasyphyllum* (leaves, stem and roots) *and* D*. abyssinica* (stem and roots) against six bacterial specious.

Extracts type		Conc	Inhibition zone diameter in (mm), including well diameter (8 mm)					
			*S.aureus*	*S. epider*	*E. faecalis*	*E. coli*	*K. pneum*	*P. aerug*
*S.dasy*	Root	100 mg/mL	25.67 ± 0.3^h^	38.33 ± 0.3^d^	29.00 ± 0.0^g^	36.33 ± 0.3^de^	29.33 ± 0.3^g^	35.67 ± 0.3^e^
		200 mg/mL	27.33 ± 0.3^h^	39.33 ± 0.3^cd^	29.33 ± 0.3^g^	37.00 ± 0.0^de^	29.67 ± 0.3^g^	36.00 ± 0.0^de^
	Stem	100 mg/mL	32.33 ± 0.3^f^	45.00 ± 0.0^a^	37.33 ± 0.3^de^	43.00 ± 0.0^bc^	37.33 ± 0.3^de^	41.00 ± 0.0^c^
		200 mg/mL	36.67 ± 0.3^e^	46.67 ± 0.3^a^	39.33 ± 0.3^cd^	43.33 ± 0.3^b^	39.33 ± 0.3^cd^	42.33 ± 0.3^c^
	Leaf	100 mg/mL	29.33 ± 0.3^g^	44.33 ± 0.3^a^	33.33 ± 0.3^ef^	39.00 ± 0.5^cd^	33.00 ± 0.0^ef^	41.00 ± 0.0^c^
		200 mg/mL	32.33 ± 0.3^f^	45.00 ± 0.0^a^	35.00 ± 1.0^de^	41.67 ± 0.3^c^	35.67 ± 0.3^e^	42.67 ± 1.2^bc^
*D. aby*	Root	100 mg/mL	22.00 ± 0.0^j^	33.00 ± 0.0^ef^	24.33 ± 0.3^i^	32.33 ± 0.3^f^	26.00 ± 0.0^h^	33.00 ± 0.0^ef^
		200 mg/mL	22.67 ± 0.3^j^	33.33 ± 0.3^ef^	24.67 ± 0.3^i^	33.67 ± 0.3^ef^	26.33 ± 0.3^h^	33.00 ± 0.0^ef^
	Stem	100 mg/mL	31.00 ± 0.0^fg^	42.00 ± 0.0^c^	32.00 ± 0.0^f^	38.33 ± 0.3^d^	34.00 ± 0.0^e^	37.00 ± 0.0^de^
		200 mg/mL	33.33 ± 0.3^ef^	43.33 ± 0.3^b^	34.67 ± 0.3^e^	40.00 ± 0.0^c^	35.33 ± 0.3^e^	39.00 ± 0.0^cd^
Tween-80		5%	8.00 ± 0.0^k^	8.00 ± 0.0^k^	8.00 ± 0.0^k^	8.00 ± 0.0^k^	8.00 ± 0.0^k^	8.00 ± 0.0^k^
Ciprofloxacin		5 μg/mL	22.33 ± 0.3^j^	34.00 ± 0.0^e^	20.00 ± 0.0^j^	32.67 ± 0.3^f^	26.00 ± 0.5^h^	34.00 ± 0.0^e^

Keynotes: Different lowercase superscript letters within each column indicate statistically significant differences among fractions (p < 0.05). Error bars represent ±SD of three replicates. Values are expressed as the mean ± SD (n = 3 or three replicates), 8 ± 0.0 = no inhibition (well diameter). Conc-Concentration, Ciprofloxacin positive control, *.S. epider- S. epidermidis, K. pneum-K. pneumoniae, P. aerug- P. aerugenosa,* Tween 80 negative control, *S. dasy.= S. dasyphyllum, D. aby. =D. abyssinica*, conc. = concentration.

A remarkable finding is the superior antibacterial performance of the *S. dasyphyllum* stem extract, particularly at 200 mg/mL, where inhibition zones reached 46.67 ± 0.3 mm against *S. epidermidis*, 43.33 ± 0.3 mm against *E. coli*, and 42.33 ± 0.3 mm against *P. aeruginosa*. This potency is significantly greater than that of the standard antibiotic Ciprofloxacin (5 μg/mL), which recorded inhibition zones of only 34.00 ± 0.0 mm, 32.67 ± 0.3 mm, and similar ranges across tested strains. This level of efficacy, despite the higher concentration of the crude extract, is indicative of a rich presence of antibacterial compounds in the stem of *S. dasyphyllum*.

The *D. abyssinica* stem extract also showed substantial activity, with inhibition zones of 43.33 ± 0.3 mm, 40.00 ± 0.0 mm, and 39.00 ± 0.0 mm at 200 mg/mL against *S. epidermidis*, *E. coli*, and *P. aeruginosa*, respectively. While slightly less potent than *S. dasyphyllum*, these results are nonetheless highly significant, especially in the context of rising resistance among Gram-negative bacteria such as *P. aeruginosa* and *E. coli*. The fact that these plant extracts perform better than Ciprofloxacin *in vitro* (despite being used in higher crude concentrations) positions them as promising candidates for further study. Interestingly, antibacterial activity was generally more pronounced at higher concentrations (200 mg/mL), confirming a dose-dependent effect, a key pharmacodynamic property. This trend reinforces the concept that active compounds within the plant matrix are effective in proportion to their availability, and supports the potential for extract optimization and purification.

A noteworthy contrast was observed with the *D. abyssinica* root extract, which showed the lowest inhibition zones, particularly against Gram-positive *E. faecalis* and *S. aureus* 22.67 ± 0.3 mm and 24.67 ± 0.3 mm at 200 mg/mL, respectively. Despite being relatively low compared to other extracts, these values remain comparable to Ciprofloxacin (20.00 ± 0.0 mm and 22.33 ± 0.3 mm, respectively), highlighting the retained antibacterial potential of even the least potent extracts. This suggests that different plant parts may accumulate different phyto-constituents, which aligns with known variations in metabolite biosynthesis across plant tissues.

Another critical observation is the stronger activity against Gram-negative bacteria, especially *E. coli* and *P. aeruginosa*, which are typically more resistant due to their complex outer membrane structure. This underscores the strength of these plant extracts and indicates the possible presence of compounds capable of penetrating or disrupting bacterial membranes. Compounds such as saponins and certain alkaloids are known for such mechanisms, and their presence could explain the observed activity. Moreover, the significant inhibition exhibited by the plant extracts compared to Ciprofloxacin (P < 0.05) is particularly important in the context of antimicrobial resistance (AMR). The superior *in vitro* performance of the crude extracts, despite being less refined, demonstrates their therapeutic relevance and potential as alternative or adjunct antibacterial agents. With Ciprofloxacin resistance on the rise globally, these findings offer a potential pathway toward the development of plant-based antimicrobials or antibiotic potentiators.

The variability in antibacterial efficacy across different plant parts and species emphasizes the importance of phytochemicals profiling and bioassay-guided fractionation to isolate and characterize the active components. It also validates traditional medicinal knowledge, as these plants have long been used in ethnomedicine for treating infections.

In summary, the methanolic extracts of *S. dasyphyllum* and *D. abyssinica*, particularly from their stem parts, demonstrated significant antibacterial activity against both Gram-positive and Gram-negative bacteria, surpassing Ciprofloxacin in several instances. The dose-dependent nature, broad-spectrum efficacy, and comparative superiority to standard antibiotics underscore the rich pharmacological potential of these plants. These findings warrant further *in vivo* studies, phytochemical investigations, and possibly the development of new antimicrobial formulations from these plant species.

##### 3.2.1.2 Minimum inhibitory concentration of the extracts

Each microorganism was tested with plant extracts starting at concentrations of 25 mg/mL and 5 mg/mL, followed by serial bi-fold dilutions down to 0.195 mg/mL to determine the MIC values. The MIC results aligned with the preliminary antimicrobial screening (on-well method) for most microorganisms. The methanol extracts from all parts of the plant leaves, stems, and roots demonstrated significant antibacterial activity against all tested organisms, as shown in Table 3. Both plant crude extracts exhibited notable antibacterial activity, demonstrating varying degrees of effectiveness against a range of bacterial strains. The minimal inhibitory concentration (MIC) values for the extracts from *S. dasyphyllum* and *D. abyssinica* ranged from 0.195 to 6.25 mg/mL. Among the tested bacterial strains *S. aureus*, *S. epidermidis*, *E. faecalis*, *E. coli*, *K. pneumoniae*, and *P. aeruginosa* the extracts showed the most significant activity against *S. epidermidis*, *E. coli*, *K. pneumoniae*, and *P. aeruginosa*, with the lowest MIC of 0.195 ± 0.0 mg/mL for the root and stem extracts of *S. dasyphyllum,* while the leaf extract demonstrated with the lowest MIC of 0.195 ± 0.0 mg/mL against *E. coli*, and MIC values of 0.260 ± 0.06 mg/mL against *S. epidermidis*, *K. pneumoniae*, and *P. aeruginosa*. In contrast, *E. faecalis* exhibited much higher MIC values, with the root extract showing the highest MIC of 6.25 ± 0.0 mg/mL, suggesting intrinsic resistance mechanisms that limit its susceptibility compared to other strains. The crude root and stem extracts of *D. abyssinica* also demonstrated strong antibacterial activity, with MIC values ranging from 0.195 to 6.25 mg/mL. The most pronounced activity was observed against *E. coli* and *P. aeruginosa*, both with MIC values of 0.195 mg/mL. Furthermore, stem extracts of *D. abyssinica* showed high antibacterial potency across all tested strains, with the highest activity recorded at an MIC of 0.260 ± 0.06 mg/mL against *S. aureus*, *E. coli*, *K. pneumoniae*, and *P. aeruginosa*. For comparison, the reference antibiotic ciprofloxacin exhibited MIC values between 0.039 ± 0.0 μg/mL and 0.312 ± 0.0 μg/mL.

**TABLE 3:** MIC values of the crude extracts against tested microorganisms.

Bacteria strains	Average MIC values from triplicate assays of plant extracts & reference drug					
	*S. dasyphyllum*			*D. abyssinica*		Ciprofloxacin
	Root	Stem	Leaf	Root	Stem	
*S. aureus*	0.391 ± 0.0^e^	0.195 ± 0.0^g^	0.260 ± 0.06^f^	3.125 ± 0.0^b^	0.260 ± 0.06^f^	0.312 ± 0.0^e^
*S. epidermidis*	0.195 ± 0.0^g^	0.195 ± 0.0^g^	0.260 ± 0.06^f^	0.781 ± 0.0^d^	0.781 ± 0.0^d^	0.156 ± 0.0^h^
*E. faecalis*	6.25 ± 0.0^a^	0.391 ± 0.0^e^	1.565 ± 0.0^c^	6.25 ± 0.0^a^	0.391 ± 0.00^e^	0.312 ± 0.0^e^
*E. coli*	0.195 ± 0.0^g^	0.195 ± 0.0^g^	0.195 ± 0.0^g^	0.195 ± 0.0^g^	0.260 ± 0.06^f^	0.078 ± 0.0^i^
*K. pneumoniae*	0.391 ± 0.0^e^	0.195 ± 0.0^g^	0.260 ± 0.06^f^	3.125 ± 0.0^b^	0.260 ± 0.06^f^	0.195 ± 0.0^g^
*P. aeruginosa*	0.195 ± 0.0^g^	0.195 ± 0.0^g^	0.260 ± 0.06^f^	0.195 ± 0.0^g^	0.260 ± 0.06^f^	0.039 ± 0.0^i^

Footnote: *S. aureus =Staphylococcus aureus, S. epidermidis =Staphylococcus epidermidis, E. faecalis = Enterococcus faecalis, E. coli =Escherichia coli, K. pneumonia= Klebsiella pneumonia, P. aeruginosa = Pseudomonas aeruginosa;* Different lowercase superscript letters within each column indicate statistically significant differences among fractions (p < 0.05). Error bars represent ±SD of three replicates.

Several factors could explain the observed differences in bacterial susceptibility to both *S. dasyphyllum* and *D. abyssinica*. The results indicate that both *S. dasyphyllum* and *D. abyssinica* possess significant antibacterial potential, with varying degrees of effectiveness across different bacterial strains. Gram-positive bacteria, particularly *S. epidermidis* and *S. aureus*, were more susceptible to the extracts, likely due to their cell wall composition. In contrast, Gram-negative bacteria, such as *E. coli, K. pneumoniae,* and *P. aeruginosa,* exhibited some resistance, which can be attributed to their outer membrane barriers, efflux pumps, and biofilm-forming abilities. Also, both plant extracts are rich in phenolic compounds and flavonoids and may work synergistically to enhance the antibacterial effects, especially against Gram-negative bacteria. The bioactive components likely acts by disrupting bacterial cell membranes, inhibiting protein synthesis, or inducing cell death through apoptosis. The presence of high concentrations of these phytochemicals may explain the observed antibacterial efficacy of the plant extracts against a wide range of bacterial strains. The higher MIC values observed for *E. faecalis* suggest intrinsic resistance mechanisms, such as biofilm formation or enzymatic degradation of antimicrobial compounds. These findings highlight the need for further research to explore the mechanisms behind the antibacterial action of these plant extracts and their potential for use in developing novel antimicrobial therapies.

The antibacterial activity of the crude extracts from both *S. dasyphyllum* and *D. s abyssinica* supports their traditional use and suggests their potential as sources for developing new antibacterial drugs. While the findings of this study are generally consistent with previous reports, earlier research on *S. dasyphyllum* has demonstrated strong antibacterial activity, particularly against *S. aureus*, *B. subtilis*, *E. coli*, and *P. aeruginosa*. This aligns with our results, especially for the methanolic extract and its fractions from the leaves of *S. dasyphyllum* (Oyinloye et al., 2023). However, the antibacterial activity observed in *D. abyssinica* differs slightly from past reports (Legesse et al., 2019). These discrepancies may be attributed to several factors, including variations in extraction methods (such as solvent type or extraction duration), the plant parts used, geographical differences, and differences in experimental protocols. These findings underscore the need for further studies with standardized methods to better understand the bioactivity of *S. dasyphyllum* and *D. abyssinica*, and to examine how environmental and methodological factors may influence their therapeutic potential.

#### 3.2.2 Antifungal activity

The antifungal activity of crude methanolic extracts from the leaves, stem, and root parts of *S. dasyphyllum* and *D. abyssinica* was evaluated against *C. albicans*, *T. mentagrophytes*, and *T. rubrum* using an agar-well diffusion assay. Extracts were tested at concentrations of 100 mg/mL and 200 mg/mL in triplicate for each plant part. The methanolic root extract of *S. dasyphyllum* exhibited potent antifungal activity, with significant inhibition zones observed against all three fungal strains. At 100 mg/mL, the root extract produced inhibition zones of 16.16 ± 0.44 mm, 16.67 ± 0.33 mm, and 10.90 ± 0.21 mm against *T. rubrum, T. mentagrophytes*, and *C. albicans*, respectively. At 200 mg/mL, the inhibition zones increased to 20.67 ± 0.33 mm, 18.50 ± 0.29 mm, and 12.83 ± 0.16 mm for the same strains. The antifungal activity was dose-dependent, with the root extract consistently outperforming the stem and leaf extracts of *S. dasyphyllum*, which showed negligible or no activity. Additionally, the antifungal effectiveness of the root extract was comparable to the positive control, Amphotericin B (32 μg/mL), against all tested fungal strains (Table 4; Figure 2).

**TABLE 4:** Inhibition zone diameter (mm) of crude extracts of *S. dasyphyllum and, D. abyssinica’s* against three fungi species.

Extract type		Concentration	Inhibition zone diameter in (mm), including 8 mm well diameter		
			*C. albicans*	*T. menta*	*T. rubrum*
*S. dasyphyllum*	root	100 mg/mL	10.90 ± 0.21^e^	16.67 ± 0.33^c^	16.16 ± 0.44^c^
		200 mg/mL	12.83 ± 0.16^d^	18.50 ± 0.29^c^	20.67 ± 0.33^b^
	Stem	100 mg/mL	8.00 ± 0.0^f^	8.00 ± 0.0^f^	8.00 ± 0.0^f^
		200 mg/mL	8.00 ± 0.0^f^	10.33 ± 0.33^e^	8.00 ± 0.0^f^
	leaf	100 mg/mL	9.00 ± 0.0^f^	8.00 ± 0.0^f^	8.00 ± 0.0^f^
		200 mg/mL	9.80 ± 0.21^e^	9.00 ± 0.0^f^	8.00 ± 0.0^f^
*D. abyssinica*	root	100 mg/mL	8.00 ± 0.0^f^	8.00 ± 0.0^f^	8.00 ± 0.0^f^
		200 mg/mL	8.00 ± 0.0^f^	8.00 ± 0.0^f^	8.00 ± 0.0^f^
	Stem	100 mg/mL	8.00 ± 0.0^f^	8.00 ± 0.0^f^	8.00 ± 0.0^f^
		200 mg/mL	8.00 ± 0.0^f^	8.00 ± 0.0^f^	8.00 ± 0.0^f^
Tween-80		5%	8.00 ± 0.0^f^	8.00 ± 0.0^f^	8.00 ± 0.0^f^
Amphotericin B		32 µg/mL	25.33 ± 0.6^a^	20.3 ± 0.6^b^	13.3 ± 0.6^d^

Key notes: Different lowercase superscript letters within each column indicate statistically significant differences among fractions (p < 0.05). Error bars represent ±SD of three replicates. Values are expressed as Mean ± SD (n = 3), 8.0 ± 0.0 = no inhibition (well diameter), Conc.-concentration, *T. menta. - T. mentagrophytes*.

The significant antifungal activity of *Solanum dasyphyllum* is likely attributed to its diverse secondary metabolites, particularly phenolic compounds and flavonoids, which are known to inhibit fungal growth by disrupting cell wall integrity and interfering with fungal enzymes (Oyinloye et al., 2023; Ng et al., 2022). The enhanced efficacy of the root extract may reflect a higher concentration of other antifungal constituents, such as alkaloids and terpenoids (Sharma et al., 2019), which are often concentrated in roots as part of the plant’s defense against soil-borne pathogens.

*Dovyalis abyssinica* extracts, previously reported to be rich in phenolic compounds and flavonoids (Baylie et al., 2024), and also demonstrated antifungal potential in this study. However, their activity was less pronounced compared to *S. dasyphyllum*, particularly in root extracts, possibly due to differences in the levels or profiles of active constituents. Nevertheless, *D. abyssinica* roots still exhibited appreciable activity, likely through synergistic interactions among phenolic compounds and other metabolites that induce oxidative stress in fungal cells by generating reactive oxygen species (ROS) (Legesse et al., 2019; Sharma et al., 2019).

Further studies into the phytochemical composition of these extracts are warranted to determine the specific compounds responsible for the observed antifungal activity. Nevertheless, the promising antifungal effects of the root extract of *S. dasyphyllum* and the potential activity of *D. abyssinica* root extracts suggest that these plants could be valuable sources of natural antifungal agents, warranting further exploration for potential therapeutic applications in treating fungal infections (Ng et al., 2022).

### 3.3 *In vitro* cytotoxic activity against MCF-7 breast cancer cells

**FIGURE 3:**
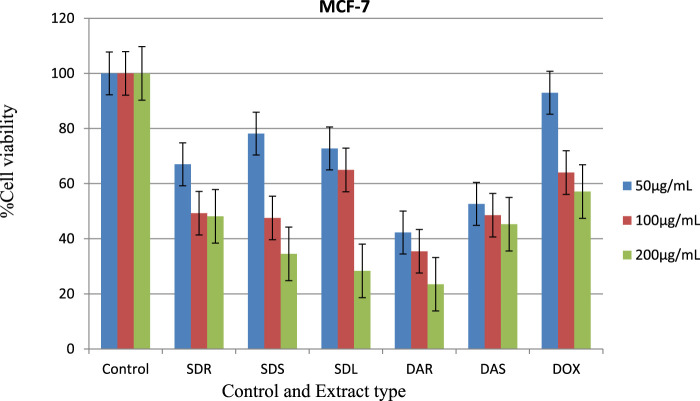
Cytotoxic effect of extracts of *S. Dasyphyllum* root, stem, leaf, and *D. abyssinica root* and *stem* against the MCF-7 human breast cancer cell line. Key notes: Extract type SDR= *S. dasyphyllum root,* SDS= *S. dasyphyllum stem,* SDL= *S. dasyphyllum leaf,* DAR=*D. abyssinica root*, DAS=*D. abyssinica stem,* and DOX= Doxorubicin.

The present study evaluated the *in vitro* cytotoxic activity of methanolic extracts from *S. dasyphyllum* and *D. abyssinica* against the human breast cancer cell line MCF-7. The results revealed a concentration-dependent decrease in cell viability across all five tested extracts SDR (root), SDS (stem), SDL (leaf), DAR (root), and DAS (stem) as presented in Table 5; Figure 3. This dose-dependent response suggests that bioactive constituents within the extracts exert measurable cytotoxic effects, supporting their potential relevance for further pharmacological evaluation.

**TABLE 5:** Cyto-toxic activity of methanol extracts of *Dasyphyllum root, stem, leaf,* and *D. abyssinica root* and *stem* against the MCF-7 human breast cancer cell line.

Extract	Conc. (µg/mL)	Absorbance at 570 nm			Average	SD	% cell viability	IC_50_ (µg)
Control	-	2.91	1.954	1.841	2.329,818	0.929,162	100.00	-
SDR	50	1.314	1.393	1.514	1.407	0.100,732	66.99772^c^	2.37
	100	1.133	0.556	1.415	1.034667	0.437,861	49.26817^e^	
	200	0.289	1.054	1.69	1.011	0.701,489	48.14122^e^	
SDS	50	1.675	1.808	1.441	1.641,333	0.185,802	78.15607^b^	1.98
	100	0.903	1.161	0.932	0.998,667	0.141,331	47.55394^e^	
	200	0.647	0.775	0.753	0.725	0.06844	34.52264^g^	
SDL	50	1.032	1.742	1.81	1.528	0.430,892	72.75943^b^	2.097
	100	0.971	1.469	1.653	1.364,333	0.352,842	64.96604^c^	
	200	0.663	0.359	0.762	0.594,667	0.21001	28.3165^h^	
DAR	50	0.957	0.978	0.543	0.826	0.24531	42.25652^f^	0.67
	100	0.396	0.708	0.538	0.547,333	0.156,209	35.45341^g^	
	200	0.85	3.682	1.119	0.9845	0.584,098	23.49253^h^	
DAS	50	1.003	1.364	0.95	1.105,667	0.225,287	52.649^d^	1.52
	100	1.033	1.00	0.952	1.019667	0.062083	48.5539^e^	
	200	0.995	0.964	0.894	0.951	0.05174	45.28417^f^	
Doxorubicin	50	2.289	2.664	2.699	2.166	1.009679	92.96863^a^	3.431
	100	3.315	1.474	2.325	1.49125	0.985,954	64.00714^c^	
	200	2.144	2.574	2.013	1.331,222	0.8008	57.13846^d^	

Key notes: Different small letters on the column for each fraction indicate significant difference (p < 0.05). Error bars represent ±SD of three replicates. Extract type SDR, *S. dasyphyllum* root*,* SDS, *S. dasyphyllum* stem, SDL, *S. dasyphyllum* leaf, DAR, *D. abyssinica* root and DAS, *D. abyssinica* stem.

Among the tested samples, *D. abyssinica* root extract (DAR) demonstrated the most potent cytotoxic activity, with an IC_50_ value of 0.67 μg/mL. This value was notably lower than that of the reference drug doxorubicin (3.431 μg/mL) under the same assay conditions. Other extracts also exhibited significant cytotoxic activity: DAS (1.52 μg/mL), SDS (1.98 μg/mL), SDL (2.09 μg/mL), and SDR (2.37 μg/mL), several of which approached or surpassed the potency of the reference compound.

These results indicate that constituents within these extracts are capable of significantly reducing MCF-7 cell viability at low concentrations. However, given the limitations of *in vitro* models, these findings remain preliminary. Additional studies, including phytochemical characterization, mechanistic assays, and *in vivo* validation, are required to assess the full pharmacological potential of these extracts.

The observed cytotoxicity may be linked to several mechanisms, including induction of apoptosis, disruption of cell cycle progression, and modulation of oxidative stress pathways mechanisms commonly associated with plant-derived cytotoxic agents (Ng et al., 2022; Sharma et al., 2019). Apoptosis, potentially via the intrinsic mitochondrial pathway, may involve the loss of mitochondrial membrane potential, cytochrome c release, and caspase activation. Cell cycle arrest may result from modulation of cyclins and cyclin-dependent kinases (CDKs), thereby halting cancer cell proliferation. Additionally, increased production of reactive oxygen species (ROS) may selectively induce oxidative damage in cancer cells, which are more vulnerable to redox imbalance.

The potent activity observed, particularly in the root extract of *Dovyalis abyssinica*, may be attributed to its rich phytochemical composition, notably its high content of phenolic compounds and flavonoids such as quercetin and kaempferol both known for their pro-apoptotic and anti-proliferative properties. The strong cytotoxic effects observed in the *D. abyssinica* root extract suggest possible synergistic interactions among these bioactive constituents (Didier et al., 2023; Samuel et al., 2024). These findings are consistent with previous reports on the cytotoxic potential of *D. abyssinica*. For instance, Emam et al. (2021) demonstrated that an 80% ethanol extract of *D. abyssinica* leaves exhibited significant cytotoxicity against HeLa and PC3 cancer cell lines. Such evidence supports the hypothesis that this species harbors compounds with broad-spectrum cytotoxic activity. Moreover, the alignment between these *in vitro* findings and the plant’s traditional medicinal use further underscores its ethnopharmacological relevance.

While the observed IC_50_ values are promising, it is important to underscore that such *in vitro* results do not equate to clinical efficacy. Natural extracts that exhibit potent cytotoxicity may serve as leads for the development of novel compounds, potentially functioning as primary agents or adjuvants to conventional chemotherapy, particularly in overcoming drug resistance. However, *in vivo* validation, pharmacokinetic profiling, and comprehensive toxicological studies are essential to determine their suitability for further development. Future research should include bioassay-guided fractionation to isolate active constituents, structural elucidation to characterize their chemical nature, and structure–activity relationship (SAR) analyses to optimize their cytotoxic potential. These efforts will be critical in advancing these extracts toward possible therapeutic applications.

## 4 Conclusion

This study provides comprehensive *in vitro* evidence supporting the pharmacological potential of methanolic extracts from Solanum dasyphyllum and Dovyalis abyssinica. Quantitative analysis revealed that the extracts are particularly rich in phenolic and flavonoid compounds, with the highest concentrations found in the root extract of D. abyssinica (143.22 ± 0.317 mg GAE/g of total phenolics and 112.36 ± 0.249 mg CE/g of total flavonoids), followed by the root extract of S. dasyphyllum. These secondary metabolites are well known for their antimicrobial and cytotoxic properties, suggesting a biochemical basis for the observed biological activities. The extracts exhibited notable antibacterial activity, with MIC values ranging from 0.195 to 6.25 mg/mL. *S. epidermidis*, *E. coli*, *K. pneumoniae*, and *P. aeruginosa* were especially susceptible, particularly to the root and stem extracts of *S. dasyphyllum* and the root extract of *D. abyssinica* (MIC = 0.195 mg/mL). Additionally, the leaf extract of *S. dasyphyllum* showed potent effects (MIC = 0.195 ± 0.0 mg/mL against *E. coli*, and 0.260 ± 0.06 mg/mL against other tested pathogens). The root extract of *S. dasyphyllum* also demonstrated antifungal activity against *T. rubrum* (inhibition zone: 20.67 mm). *In vitro* cytotoxicity assays against MCF-7 breast cancer cells revealed concentration-dependent effects for all extracts, with *D. abyssinica* root extract showing the greatest potency (IC_50_ = 0.67 μg/mL), exceeding that of doxorubicin under the same conditions. These cytotoxic responses may involve apoptosis induction, modulation of oxidative stress, or interference with cell cycle regulation, though these mechanisms remain to be confirmed. The findings support the pharmacological potential of *S. dasyphyllum* and *D. abyssinica* extracts as promising candidates for antimicrobial and cytotoxic drug discovery. However, further studies involving *in vivo* validation, toxicity profiling, mechanistic investigation, and bioassay-guided isolation of active compounds are essential to substantiate these results and facilitate the development of safe, effective phytotherapeutics.

## Data Availability

The original contributions presented in the study are included in the article Supplementary Material, further inquiries can be directed to the corresponding author.
