# Supplementary material for: In vitro antimicrobial and cytotoxic evaluation of leaf, root, and stem extracts of Solanum dasyphyllum and root and stem extracts of Dovyalis abyssinica
Source: Front Pharmacol. 2025 Jun 26;16:1529854. doi: 10.3389/fphar.2025.1529854 (PMC12243875; doi:10.3389/fphar.2025.1529854)
Supplement: Supplementary file 1 [file DataSheet1.pdf]

Supplementary Materials

In Vitro Antimicrobial and Cytotoxic Evaluation of Leaf, Root, and Stem Extracts of *Solanum dasyphyllum* and Root and Stem Extracts of *Dovyalis abyssinica*

Dereilo Bekere Belitibo[1,2*], Asfaw Meressa[1], Temesgen Negassa[1], Abiy Abebe[1], Sileshi Degu[1], Milkyas Endale[1] Frehiwot Teka Assamo[1], Temesgen Abdisa Ayana[3], Getahun Tadesse Gurmessa,[2] and Negera Abdissa[1,2]

1. Traditional and Modern Medicine Research and Development Directorate, Armauer Hansen Research Institute, P. O. Box: 1005, Addis Ababa, Ethiopia
2. Department of Chemistry, College of Natural and Computational Sciences, Wollega University, Nekemte, Ethiopia
3. Department of Chemistry, College of Natural Sciences, Jimma University, Jimma, Ethiopia

* Correspondence should be addressed to Dereilo Bekere Belitibo, E-mail: derilobakere@gmail.com

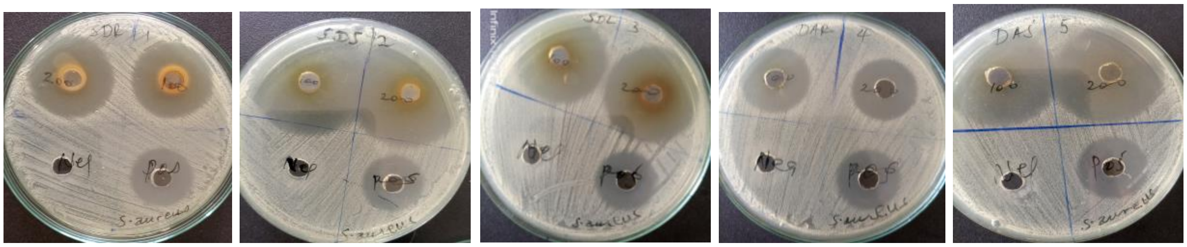

S.aureus

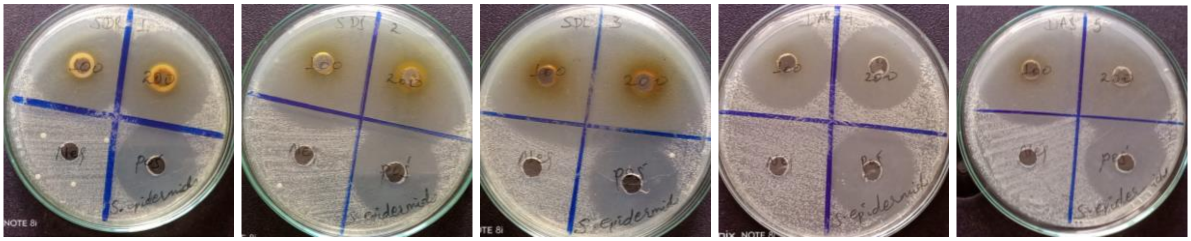

S. epidermidis

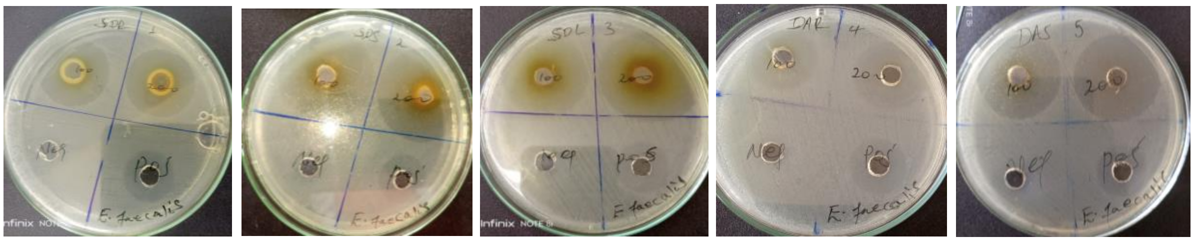

E. faecalis

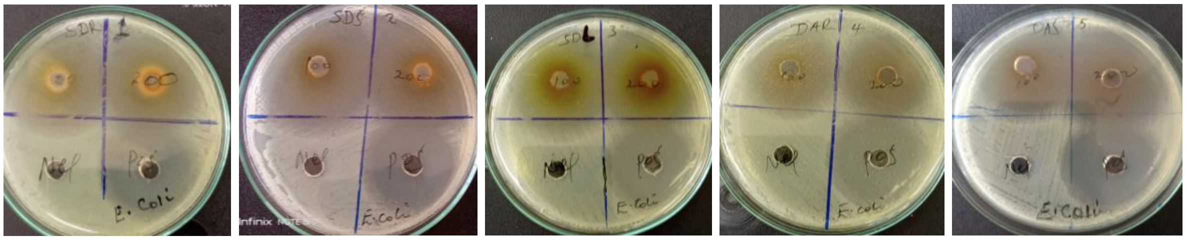

E. coli

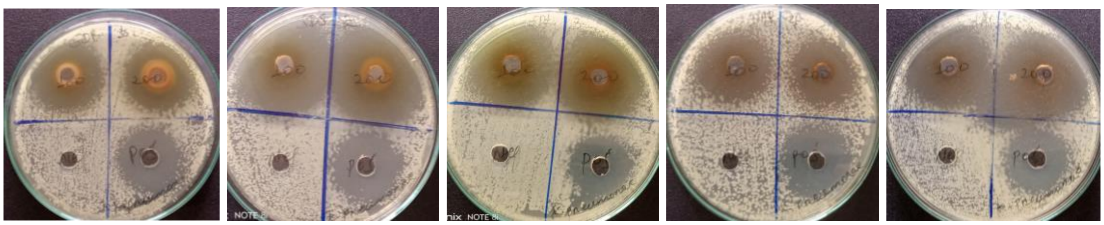

K. pneumoniae

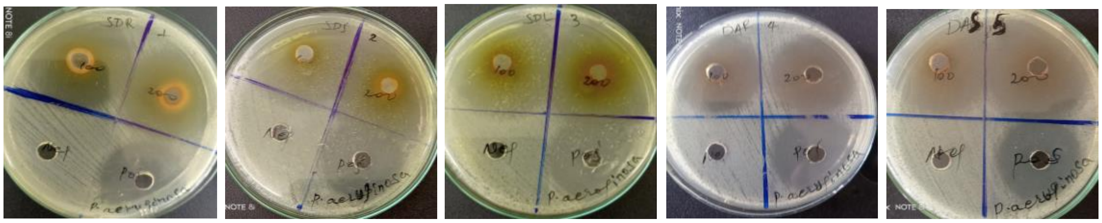

P. aerugenosa

Key notes: Extract type SDR= *S. dasyphyllum root,* SDS= *S. dasyphyllum stem,* SDL= *S. dasyphyllum leaf,* DAR=*D. abyssinica root* and DAS=*D. abyssinica stem*

Figure. **S1.** Antibacterial sctivity screening of *S. dasyphyllum* and *D. abyssinica* extracts from soot, stem, and leaf against *S. aureus, S. epidermidis, E. faecalis, E. coli, K. pneumoniae*, and *P. aeruginosa* at 100 and 200 mg/ml Concentrations

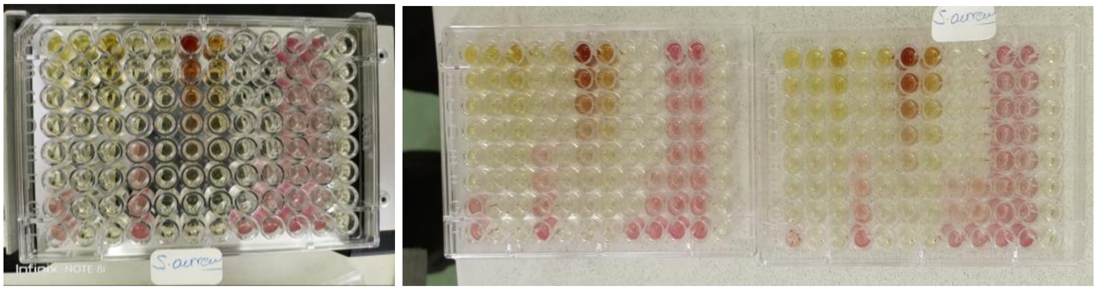

S.aureus

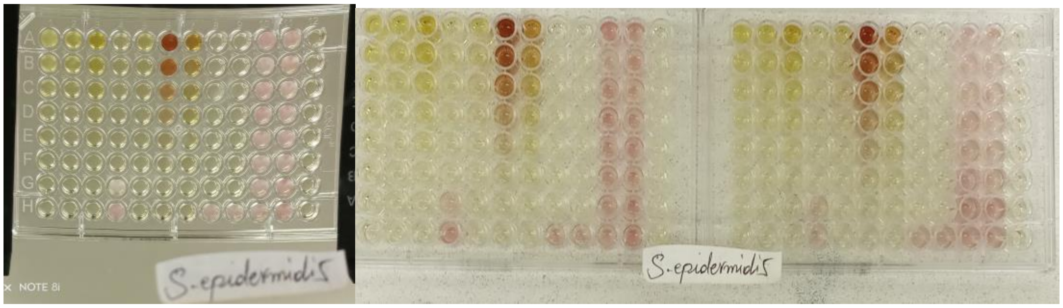

S. epidermidis

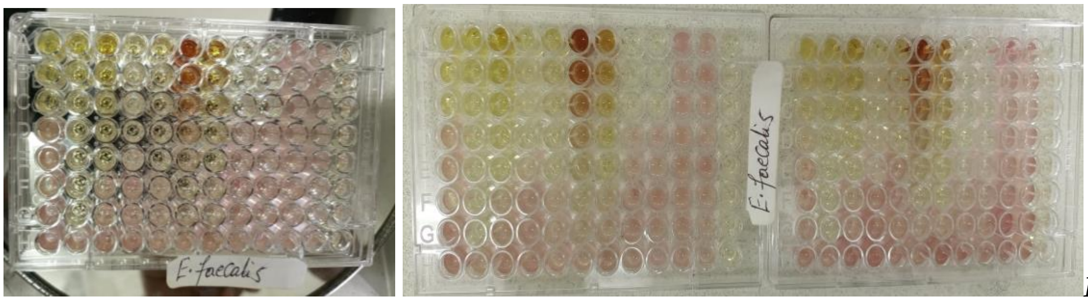

E. faecalis

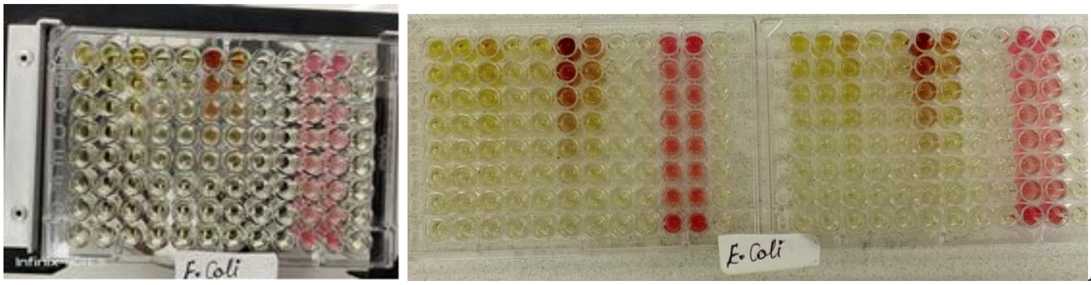

E. coli

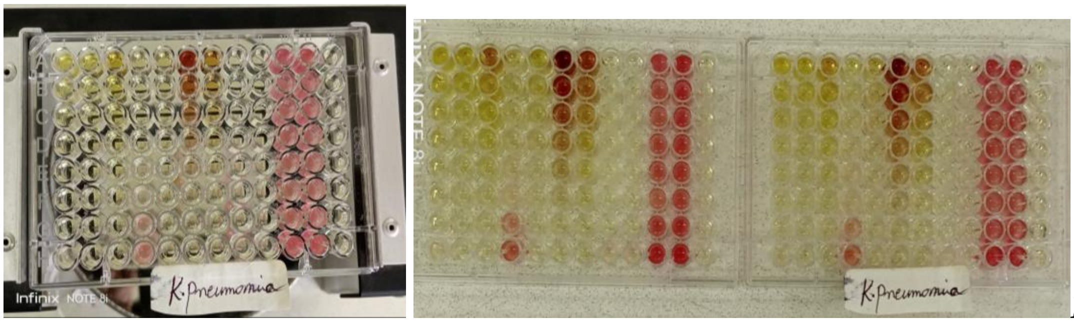

K. pneumoniae

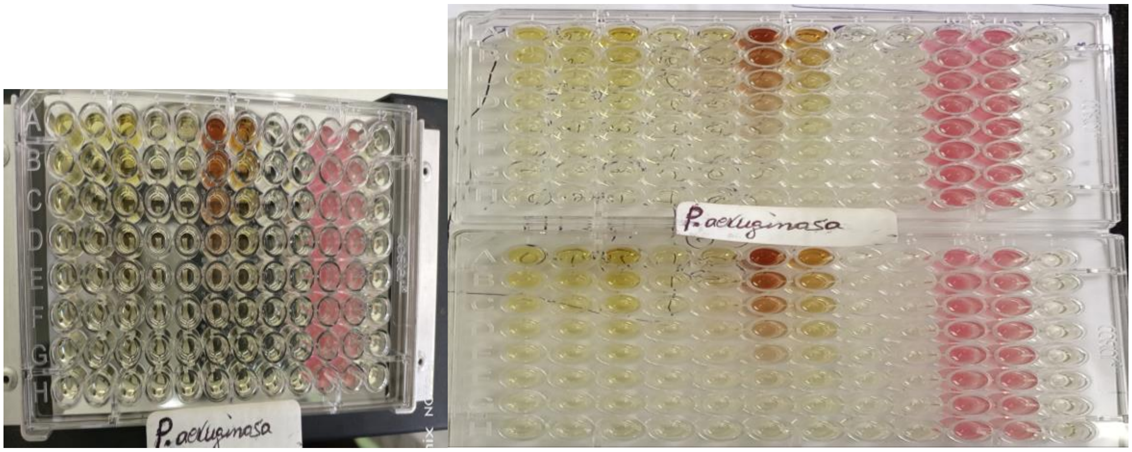

P. aerugenosa

Keynotes: For the 96-well plate, begin with rows 1 to 7 for the samples, rows 8 and 9 for the positive control (Ciprofloxacin), rows 10 and 11 for the negative and growth controls, and row 12 for the sterility control. Rows 1 to 5 correspond to the root, stem, and leaf of *S. dasyphyllum*, and the root and stem of *D. abyssinica* were the target samples being tested.

Figure S2. Photo of Antibacterial activity by using the micro-broth dilution method MIC values of the crude extracts against tested microorganisms

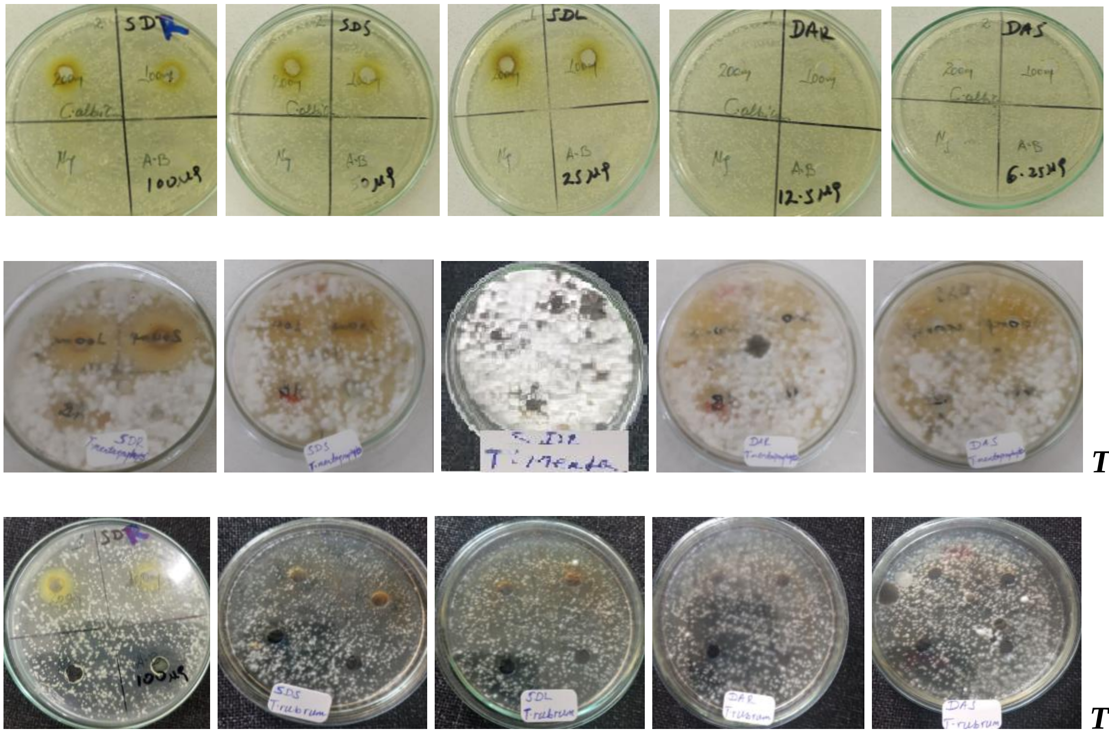

C. albicans

T. mentagrophytes

T. rubrum

Key notes: Extract type SDR= *S. dasyphyllum root*, SDS= *S. dasyphyllum stem,* SDL= *S. dasyphyllum leaf,* DAR=*D. abyssinica root* and DAS=*D. abyssinica stem*

Figure.**S3.** antifungal sctivity screening of *S. dasyphyllum* and *D. abyssinica* extracts from soot, stem, and leaf against *C. albicans*, *T. mentagrophytes*, and *T. rubrum* at 100 and 200 mg/ml Concentrations
